# Human antibodies against the myelin oligodendrocyte glycoprotein can cause complement-dependent demyelination

**DOI:** 10.1186/s12974-017-0984-5

**Published:** 2017-10-25

**Authors:** Patrick Peschl, Kathrin Schanda, Bleranda Zeka, Katherine Given, Denise Böhm, Klemens Ruprecht, Albert Saiz, Andreas Lutterotti, Kevin Rostásy, Romana Höftberger, Thomas Berger, Wendy Macklin, Hans Lassmann, Monika Bradl, Jeffrey L. Bennett, Markus Reindl

**Affiliations:** 1 Clinical Department of Neurology, Medical University of Innsbruck, Innsbruck, Austria; 2 Department of Neuroimmunology, Center for Brain Research, Medical University Vienna, Vienna, Austria; 3 Department of Cell and Developmental Biology, University of Colorado School of Medicine, Aurora, CO USA; 4 Department of Neurology, Charité-Universitätsmedizin Berlin, Berlin, Germany; 5 Service of Neurology, Department of Neurology, Hospital Clinic, Institut d’Investigacions Biomédiques August Pi i Sunyer (IDIBAPS) University of Barcelona, Barcelona, Spain; 6 Neuroimmunology and Multiple Sclerosis Research, Department of Neurology, University Hospital Zurich and University of Zurich, Zurich, Switzerland; 7 Department of Pediatric Neurology, Witten/Herdecke University, Children’s Hospital Datteln, Datteln, Germany; 8 Institute of Neurology, Medical University of Vienna, Vienna, Austria; 9 Departments of Neurology and Ophthalmology, Program in Neuroscience, School of Medicine, University of Colorado, Aurora, CO USA

**Keywords:** Myelin oligodendrocyte glycoprotein, MOG, Antibodies, Organotypic slice culture, EAE, Neuromyelitis optica spectrum disorders

## Abstract

**Background:**

Antibodies to the myelin oligodendrocyte glycoprotein (MOG) are associated with a subset of inflammatory demyelinating diseases of the central nervous system such as acute disseminated encephalomyelitis and neuromyelitis optica spectrum disorders. However, whether human MOG antibodies are pathogenic or an epiphenomenon is still not completely clear. Although MOG is highly conserved within mammals, previous findings showed that not all human MOG antibodies bind to rodent MOG. We therefore hypothesized that human MOG antibody-mediated pathology in animal models may only be evident using species-specific MOG antibodies.

**Methods:**

We screened 80 human MOG antibody-positive samples for their reactivity to mouse and rat MOG using either a live cell-based assay or immunohistochemistry on murine, rat, and human brain tissue. Selected samples reactive to either human MOG or rodent MOG were subsequently tested for their ability to induce complement-mediated damage in murine organotypic brain slices or enhance demyelination in an experimental autoimmune encephalitis (EAE) model in Lewis rats. The MOG monoclonal antibody 8-18-C5 was used as a positive control.

**Results:**

Overall, we found that only a subset of human MOG antibodies are reactive to mouse (48/80, 60%) or rat (14/80, 18%) MOG. Purified serum antibodies from 10 human MOG antibody-positive patients (8/10 reactive to mouse MOG, 6/10 reactive to rat MOG), 3 human MOG-negative patients, and 3 healthy controls were tested on murine organotypic brain slices. Purified IgG from one patient with high titers of anti-human, mouse, and rat MOG antibodies and robust binding to myelin tissue produced significant, complement-mediated myelin loss in organotypic brain slices, but not in the EAE model. Monoclonal 8-18-C5 MOG antibody caused complement-mediated demyelination in both the organotypic brain slice model and in EAE.

**Conclusion:**

This study shows that a subset of human MOG antibodies can induce complement-dependent pathogenic effects in a murine ex vivo animal model. Moreover, a high titer of species-specific MOG antibodies may be critical for demyelinating effects in mouse and rat animal models. Therefore, both the reactivity and titer of human MOG antibodies must be considered for future pathogenicity studies.

**Electronic supplementary material:**

The online version of this article (10.1186/s12974-017-0984-5) contains supplementary material, which is available to authorized users.

## Background

In the last decades, several studies reported that autoantibodies against the myelin oligodendrocyte glycoprotein (MOG) are associated with inflammatory demyelinating diseases of the central nervous system (CNS), such as acute disseminated encephalomyelitis (ADEM), monophasic or recurrent isolated optic neuritis (ON) or transverse myelitis (TM), pediatric multiple sclerosis (MS), aquaporin-4 (AQP4)-seronegative neuromyelitis optica spectrum disorders (NMOSD), and *N*-methyl d aspartate receptor (NMDAR) encephalitis with overlapping demyelinating syndromes [1–16]. Although MOG antibodies are rarely found in MS, MOG-seropositive patients show neuropathological features that are similar to MS pattern II pathology with demyelinated lesions, preserved axons and complement-mediated pathology [17–20]. MOG is a very well-studied antigen in experimental autoimmune encephalomyelitis (EAE), which is often used as a model for MS. These animal models have been developed using active immunization with MOG, transfer of MOG-specific T cells and antibodies, and mice transgenic for MOG-specific T and B cell receptors [21–27]. In EAE, it was demonstrated that MOG antibodies are strongly pathogenic only after inflammation and blood-brain barrier leakage induced by encephalitogenic T cells. Co-transfer of the mouse monoclonal anti-MOG antibody 8-18-C5 leads to severe complement-dependent lesions in a myelin basic protein (MBP)-induced EAE model in Lewis rats [21, 22, 28] and in vitro brain cell cultures [29].

Nevertheless, the effects of human MOG (hMOG) antibodies and their exact role in demyelinating diseases are still not fully understood. While hMOG antibodies can initiate both complement-dependent cytotoxicity (CDC) and antibody-dependent cellular cytotoxicity (ADCC) in an Fc-dependent manner [30–34], in vitro studies have demonstrated MOG antibody binding causes disturbances of thin filaments and microtubule cytoskeleton in oligodendrocytes [35] and cellular cytotoxic effects [2] independent of effector function. Previous in vivo studies have indicated that MOG antibodies trigger the activation of MOG-specific T cells by facilitating opsonization and accumulation in antigen-presenting cells in the CNS and periphery. This process then fosters T cells to recognize their autoantigen and activate in an Fc-dependent manner [36, 37]. Administration of hMOG antibodies into a MBP-T cell-mediated EAE model in Lewis rats resulted in minor demyelination and axonal loss in the absence of excessive complement activation [38], and intracerebral injection of hMOG-IgG into murine brain resulted in complement-independent reversible demyelination and axonal loss [39].

It has become increasingly clear that only MOG antibodies recognizing correctly folded and glycosylated MOG are disease relevant. Although MOG is highly conserved between different species, previous studies have shown that single amino acid exchanges between mouse MOG (mMOG) and hMOG led to a loss of binding ability of most hMOG antibodies to mMOG [40]. These experimental observations and the polyclonal nature of serum IgG reinforce that not all hMOG autoantibodies will be reactive to rodent MOG [40, 41]. To our knowledge, these species-specific recognition patterns have not been systematically evaluated when analyzing the pathogenicity of human MOG antibodies. Therefore, our study aimed to investigate whether hMOG autoantibodies carefully selected for binding to both hMOG and rodent MOG can induce demyelinating pathology in rodents. We screened hMOG-reactive patient sera for reactivity to mMOG and rat MOG (rMOG), analyzed binding patterns in different brain tissues, and selected a set of high and low titer IgG samples with different binding patterns to rodent MOG to perform ex vivo and in vivo studies for demyelinating activity.

## Methods

### Patients

Serum samples were collected at the Clinical Department of Neurology, Medical University of Innsbruck between 2008 and 2016 and stored at − 80 °C until use. This study was approved by the Ethical Committee of the Medical University of Innsbruck (study numbers AM3041A and AM4059) and by the Ethic Committees of the Hospital Clinic of Barcelona (2010/5680), Charite University Medicine Berlin (EA1/131/09), and University Hospital Zürich (KEK-Nr. 2013-0001). All patients or their caregivers gave informed written consent.

Serum samples were collected from 80 hMOG antibody positive, 20 hMOG antibody negative individuals, and 4 healthy controls: 48 ADEM patients, 4 multiphasic demyelinating encephalomyelitis (MDEM) patients, 13 NMOSD patients, 21 patients with clinically isolated syndrome (CIS; 13 ON, 6 myelitis and 2 multifocal), 7 recurrent ON patients, and 7 MS patients (Table 1). NMOSD was diagnosed by the 2015 International Panel for NMO Diagnosis Criteria, ADEM was diagnosed according to the criteria of the International Pediatric MS Study Group, and MS was diagnosed according to the 2010 revised McDonald criteria [42–44].

**Table 1:** Demographic and clinical data and antibody reactivity of patients included in this study according to the human MOG antibody status

	hMOG antibody negative^a^	hMOG antibody positive^a^	*P* value
Number of patients/samples	20	80	
hMOG antibody titer [1:]^b^		1280 (160–20,480)	
Females	9 (45%)	37 (46%)	0.999^c^
Age (years)^b^	12.4 (3.5–44.8)	7.0 (0.2–71.1)	0.020^d^
Pediatric patients	15 (75%)	69 (83%)	0.303^c^
Disease duration (years)^b^	0.3 (0–19.7)	0.1 (0–15.4)	0.038^d^
Clinical diagnosis at sampling:			
ADEM	11 (55%)	37 (46%)	< 0.001^e^
CIS-ON	0 (0%)	13 (16%)	
CIS-LETM	1 (5%)	5 (6%)	
CIS-multifocal	0 (0%)	2 (3%)	
MDEM	0 (0%)	4 (5%)	
NMOSD	1 (5%)	12 (15%)	
Recurrent ON	0 (0%)	7 (9%)	
MS	7 (35%)	0 (0%)	
Recurrent course at sampling	7 (35%)	17 (21%)	0.243^c^
Reactive with mouse MOG^a^	0 (0%)	48 (60%)	< 0.001^c^
Median titer (range)		640 (160–20,480)	
Reactive with rat MOG^a^	0 (0%)	14 (18%)	0.066^c^
Median titer (range)		1280 (160–5120)	
Reactivity with brain tissue:			
Antibody binding to human myelin	0 (0%)	70 (88%)	< 0.001^c^
Antibody binding to mouse myelin	1 (5%)	27 (34%)	0.011^c^
Thereof mMOG reactive	0/1 (0%)	25/27 (93%)	
Thereof rMOG reactive	0/1 (0%)	11/27 (41%)	
Therof mMOG or rMOG reactive	0/1 (0%)	25/27 (93%)	
Antibody binding to rat myelin	4 (20%)	24 (30%)	0.578^c^
Thereof mMOG reactive	0/4 (0%)	23/24 (96%)	
Thereof rMOG reactive	0/4 (0%)	14/24 (58%)	
Therof mMOG or rMOG reactive	0/4 (0%)	23/24 (96%)	

*Abbreviations*: *hMOG* human myelin oligodendrocyte glycoprotein, *mMOG* mouse MOG, *rMOG* rat MOG, *ADEM* acute demyelinating encephalomyelitis, *CIS* clinically isolated syndrome, *ON* optic neuritis, *LETM* longitudinally extensive transverse myelitis, *MDEM* multiphasic demyelinating encephalomyelitis, *NMOSD* neuromyelitis optica spectrum disorders, *MS* multiple sclerosis

^a^Positive for antibodies to hMOG, mMOG, or rMOG at a cutoff ≥ 1:160

^b^Median (range). Significance of group differences was calculated using

^c^Fisher’s exact test

^d^Mann-Whitney *U* test

^e^Chi square test

### Animals

Animal experiments on Lewis rats were approved by the Ethical Committee of the Medical University of Vienna (BMWF-66.009/0196-WF/V/3b/2015). Rats were obtained from Charles River Wiga (Sulzfeld, Germany) and housed in the Decentral Facilities of the Institute for Biomedical Research, Vienna under standard conditions until animals reached the age of 7–9 weeks.

Transgenic PLP-EGFP mice were housed in at the University of Colorado in accordance with University of Colorado IACUC policy for animal use, which is in compliance with the NIH Guide for the Care and Use of Laboratory Animals. Pups aged P10–P12 were used for cerebellar slice cultures.

### Screening for serum reactivity to human, mouse, and rat MOG on a live cell-based assay

One hundred samples were analyzed for antibodies against hMOG, rMOG, and mMOG by a live cell-based assay (CBA) as described previously [6, 45]. Briefly, HEK-293A cells (ATCC, LGC Standards GmbH, Wesel, Germany) were transiently transfected with Fugene HD transfection reagent (Promega Corporation, Madison, WI, USA) using the pEGFP-N1 vector (Clontech Laboratories, Mountain View, CA, USA) expressing either hMOG, mMOG, or rMOG fused to EGFP at the C terminus. Cells were blocked with goat IgG (Sigma-Aldrich, St. Louis, MO, USA). For pre-screening, samples were diluted 1:20 and 1:40 in PBS/10%FCS (Sigma-Aldrich) and incubated for 1 h at 4 °C. Bound antibodies were detected using Cy™3-conjugated goat anti-human IgG antibody (H+L, Jackson ImmunoResearch Laboratory, West Grove, PA, USA) for 30 min at room temperature (RT). DAPI staining (Sigma-Aldrich) was used to exclude dead cells. Cell bound antibodies were determined using a fluorescence microscope (Leica DMI, 4000B, Wetzlar, Germany). Seropositivity was evaluated by two blinded investigators (PP, KS), and antibody titers were determined by endpoint titrations. Specificity of this assay was tested with hMOG-negative, AQP4-positive samples, and healthy controls [41]. Non-specific background binding can be clearly distinguished from MOG-positive antibody signal.

Immunoglobulin G subclasses were evaluated using mouse anti-human IgG1, IgG2, IgG3, and IgG4 antibodies (Thermofisher Scientific, Waltham, MA, USA) and stained with Alexa 594 goat anti mouse IgG (H+L) (Thermo Fisher Scientific).

### Binding capacities of human MOG antibodies to human, rat, and mouse brain tissue

All serum samples were analyzed for IgG antibodies directed against myelin antigens by immunohistochemistry (IHC) on mildly fixed, snap-frozen rat, mouse, or human postmortem brain tissue as described previously [46]. Briefly, human cerebellar autopsy, taken about 24-h postmortem, or frontal lobe biopsy were mildly fixed in 4% PFA for 1 h at 4 °C and subsequently cryoprotected using 40% Sucrose/PBS (Merck, Darmstadt, Germany) at 4 °C for 2–3 days. Tissue was imbedded in freezing medium (O.C.T™, Tissue-Tek®, Sakura, Alphen aan den Rijn, Netherlands) and snap-frozen in liquid nitrogen pre-chilled methylbutan (Sigma-Aldrich). Rats and mice were sacrificed either with CO_2_ or cervical dislocation, sagitally sectioned, and treated like human brain tissue. Rodent and human frozen tissue was sectioned into 7-μm-thick slices and blocked for 1 h at RT using 5% normal goat serum (NGS) and 1% bovine serum albumin (BSA) in 0.05% PBS-Tween. Serum samples were pre-adsorbed overnight on a shaker with 10 mg/100 μl Calf Liver Powder (produced in house according to Coons et al. [47]) in 1%NGS/1%BSA/PBS. Pre-adsorbed serum was added to the blocked slices in a final dilution of 1:100 in 1%NGS/1%BSA/0.05%Tween/PBS and incubated over night at 4 °C. Bound serum IgG was detected using Alexa Fluor, 488 conjugated AffiniPure F(ab)_2_ fragment goat anti human IgG (Jackson ImmunoResearch, West Grove, PA, USA) in 1%NGS/1%BSA/0.05%Tween/PBS. Afterwards, slides were mounted with IF media (Dianova, Hamburg, Germany) and analyzed with a fluorescence microscope (Leica DMI4000). To deplete hMOG-specific antibodies, serum IgG was pre-adsorbed with hMOG-transfected HEK 293 cells.

### Production of monoclonal mouse and humanized 8-18-C5

Mouse monoclonal MOG antibodies (isotype IgG2B) were produced with the 8-18-C5-specific hybridoma cell line (kindly provided by Christopher Linington, University of Aberdeen, UK) and cultured in Hybridoma-SFM Medium (Gibco; Thermo Fisher Scientific). Supernatant was collected and cells split frequently to ensure optimal growth conditions. Humanized 8-18-C5 was produced by cloning the coding sequence of the variable region of mouse 8-18-C5-specific Kappa-light (8-18-C5-vLC) and heavy F.ab 1 chain (8-18-C5-vHC) (kindly provided by Klaus Dornmair, LMU, Munich, Germany) into the vector pFUSE-CHIg-hG1 (encoding the constant region of the heavy chain) and pFUSE2-CLIg (encoding the constant region of the light chain) (InvivoGen, San Diego, CA, USA), respectively. Vectors, containing 8-18-C5-specific heavy and light chains, were transfected simultaneously into HEK 293FT cells cultured in Freestyle™ Expression Medium (Gibco, Thermo Fisher scientific), using Polyethylenimine (PEI) (1 mg/ml, Sigma-Aldrich) as a transfection agent. Double transfected cells were selected and cultured in growth medium containing Blasticidin (10 μg/ml, Gibco, Thermo Fisher scientific), for pFUSE2-CLIg and Zeocin (400 μg/ml, Invitrogen, Thermo Fisher scientific), for pFUSE-CHIg-hG1. Supernatant was collected and cells split frequently to ensure optimal growth conditions.

### Antibody purification

Human antibodies from sera and therapeutic plasma exchange material as well as monoclonal 8-18-C5 were purified using Protein G Sepharose (GE Healthcare, Munich, Germany) beads and adjusted to a concentration of 5 and 10 mg/ml with 100 kDa centrifugation filter columns (Amicon Ultra, Merck, Darmstadt, Germany) for murine cerebellar slice culture and the EAE injection model, respectively. After sterile filtration, purity was checked by SDS-PAGE and functionality was tested using the live cell-based anti-MOG assay.

### Ex vivo mouse organotypic cerebellar slice model

Cerebella were dissected from P10-P12 old PLP-EGFP mice, cut sagittally into 300-μm slices, and cultured on 0.4-μm membrane inserts (Merck Millipore, Billerica, MA, USA) for 7–9 days in sterile minimum essential media containing 25% Hank’s balanced salt solution and 25% inactivated horse serum, as described previously [48]. Media was changed every 3 days to maintain culture integrity. Slice integrity was observed by excluding tissue with necrotic holes and limited folia formation. Prior to antibody treatment, media was exchanged with serum-free media (Neurobasal-A media (Life technologies), enriched with B27, 2 mM l-Glutamine, 2% HEPES, and 18 mM d-Glucose (Sigma-Aldrich)). Human antibodies were diluted to a final concentration of 1 mg/ml in serum-free media with or without 10% human sera containing complement (Complement Technology, Inc., Tyler, TX, USA) and slices were subsequently incubated for 72 h at 37 °C, 5% CO_2_. Monoclonal 8-18-C5 was used in a concentration of 10 μg/ml. Each IgG sample was tested on healthy murine organotypic brain slices in at least two repetitions and two random cerebellar folia were imaged by confocal microscopy. Myelin status was evaluated by a MBP rating scale (median myelin score; MMS) (0 = healthy myelin with well-preserved myelin sheaths around myelinated axons; 1 = slight MBP degradation and myelin disruption; 2 = partial myelin loss, intermediate to severe MBP damage; 3 = almost complete loss of myelin along myelinated axons and severe MBP loss). Variations within the range of MMS were caused by unspecific degradation processes during cultivation of the cerebellar preparations in some of the slices, which could be diminished by repeated testing and median calculations.

At the end of the experiment, slices were fixed with 4% PFA in PBS and processed for IHC. Briefly, slices were permeabilized in 10% Triton X-100 in PBS, blocked with 5% normal donkey serum (NDS) in 0.3% PBS-Triton X-100 for 1 h at RT and then incubated with primary antibodies in blocking solution: mouse anti-MBP (1:1000) (Covance, Princeton, NJ, USA) and an axonal marker, either chicken anti-neurofilament (1:10,000) (Covance) or/and chicken anti-calbindin (1:1000) (Neuromics). Following primaries, slices were washed, stained with secondary antibodies conjugated to Alexa Fluor (Jackson Immuno), washed with PBS, and mounted with Fluoromount-G (Southern Biotech, Birmingham, AL, USA). Due to potential competition between patient and commercial MOG antibodies, MOG staining was omitted and overall myelin integrity was evaluated using MBP staining [49, 50].

### MOG-IgG EAE

MBP-specific T cells were established as described earlier [51]. EAE was induced by passive transfer of freshly activated MBP-T cells (2 × 10^6^) into 7–9-weeks old Lewis rats. Weight loss and changes in the EAE score [52] in all animals indicated the first clinical signs of EAE. Four days post-treatment, animals were injected intraperitoneally with 5 mg monoclonal 8-18-C5 or isotype control, 10 or 40 mg purified IgG from MOG-positive subjects, negative control samples and normal human IgG (Subcuvia™) (Baxter, Vienna, Austria). As an additional control, animals were injected with patient IgG without prior EAE induction (PBS transfer only). Animals were sacrificed with CO_2_ 24 or 48 h post injections. Blood samples were taken by cardiac puncture, and animals were subsequently perfused with 4% PFA. Spinal cords were dissected and immersed for another 24 h in PFA and routinely embedded in paraffin. Tissue sections were stained with hematoxylin and eosin, Kluver-Barrera (myelin), anti-MOG, anti-C9neo (complement), anti-CD3 (T cells), and anti-ED1 (macrophages). Spinal cord sections of the rats were quantified according to the following score: Demyelination score (DMS) 0 = no lesions/slice, DMS 1 = one minor lesion/slice (0.05–0.2 mm diameter), DMS 2 = two minor lesions/slice or one medium lesion (> 0.2 mm diameter), DMS 3 = two or more medium lesions/slice, and DMS 4 = extensive loss of myelin.

### Statistical analysis

Statistical analysis was done using IBM SPSS software (release 24.0, IBM) or GraphPad Prism 7 (GraphPad). Between-group comparisons were performed with Mann-Whitney *U* test, Friedman test, Fisher’s exact test, and chi-square test. Statistical significance was defined as two-sided *p* value < 0.05, and Bonferroni corrections were applied for multiple comparisons when appropriate.

## Results

### Limited human MOG-positive sera react with both murine and rat MOG in a live cell-based assay

**Fig. 1:**
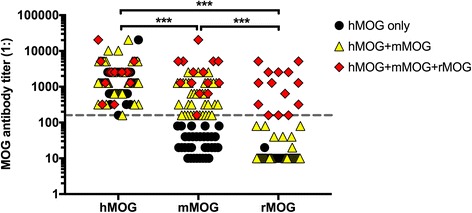
Serum antibody titer levels to human, mouse, and rat MOG by live cell-based assay. The cutoff titer value of 1:160 is indicated by a dashed horizontal line. Individual antibody titers are shown as black circles (reactive to hMOG only), yellow triangles (reactive to hMOG+mMOG) or red squares (reactive to hMOG+mMOG+rMOG). Antibody titers were compared using a non-parametric test (Friedman’s test with Dunn’s multiple comparisons test, overall *p* value < 0.001). ***p < 0.001

We screened 80 hMOG-positive (titer ≥ 1:160) serum and plasma exchange samples (median titer 1:1280, range 160–20,480) for IgG-binding to mMOG and rMOG using a CBA (Table 1, Fig. 1, and Additional file 1). To exclude non-specific binding the high-titer cutoff value of ≥ 1:160 was applied. Forty-eight samples (60%) were positive for mMOG and only 14 samples (18%) for rMOG (Table 1 and Fig. 1). Samples positive to rodent MOG were either reactive to mMOG only (34/48, 71%) or both, mMOG and rMOG (14/48, 29%); none of them bound to rMOG only. No significant association between antibody binding to mMOG or rMOG and clinical diagnosis was observed (Additional files 2 and 3). Neither the 20 hMOG-negative controls nor the 4 healthy controls were positive for rMOG or mMOG at a cutoff ≥ 1:160 (Table 1). Twenty-seven follow-up samples from 25 patients did not reveal any change in species-specific reactivity (data not shown).

### Immunohistochemistry with human MOG antibodies on human, mouse, and rat brain tissue is less sensitive than CBA

**Fig. 2:**
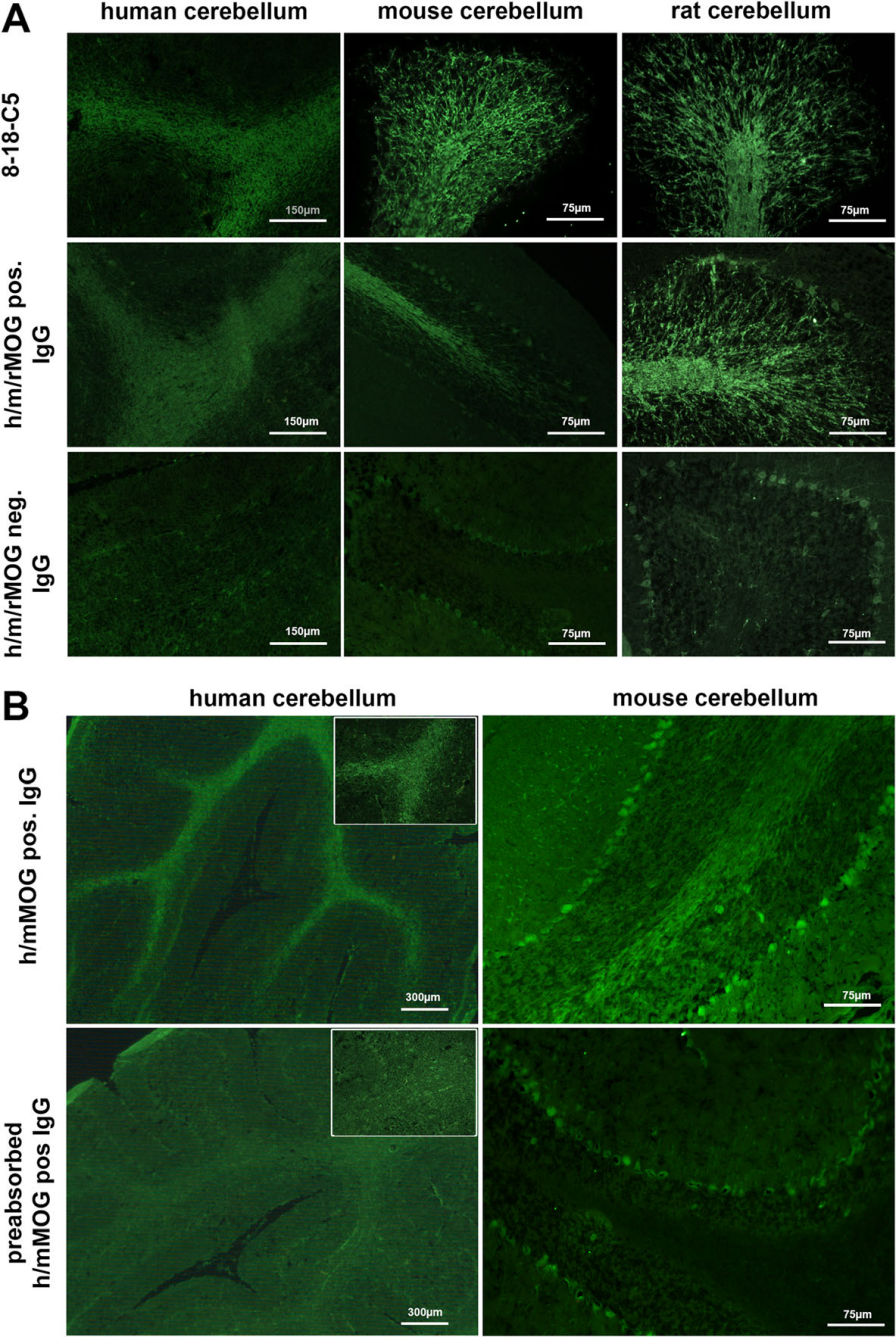
**a** Myelin staining with serum from human samples and monoclonal 8-18-C5 on human, mouse, or rat cerebellum. White matter tracts and folia were clearly stained with MOG antibody-positive serum samples and 8-18-C5 but not in MOG antibody-negative control samples. Sections were magnified at ×20 (mouse/rat tissue) and at ×10 (human tissue). **b** Serum samples reactive to human and mouse MOG bind to both human and mouse cerebellum sections. Pre-adsorbed samples show a significant reduction in MOG-specific myelin staining. Sections were magnified at ×2.5 (human tissue) and at ×20 (mouse tissue). Inlays show higher magnification (10×) of human white matter tracts

We next evaluated the binding of MOG-seropositive serum samples to rodent and human brain tissue. The criterion for positive tissue reactivity was a clear myelin staining in the white matter tract of the cerebellum, corpus callosum, or internal capsule, clearly distinguishable from the regular background of healthy controls (Fig. 2). Only 27/80 hMOG-reactive samples (34%) showed positive staining on mouse tissue (Table 1). Twenty-five of them were positive (cutoff ≥ 1:160) for mMOG in the CBA, and two had only low titers to mMOG (1:40) (Table 1 and Additional file 2). Similar results were seen when hMOG-positive samples were tested on rat tissue. Altogether, 24/80 hMOG-positive samples (30%) showed a positive staining on rat tissue (Table 1). Fourteen (58%) of them were reactive to rMOG at a cutoff of 1:160, 4 had low titers to rMOG (1:20–1:80), and 6 were tested negative for rMOG in the CBA (Table 1 and Additional file 3). Two samples with no reactivity to rodent MOG bound to mouse tissue and one sample to rat tissue.

All hMOG antibody-positive samples were also tested on human brain tissue. Seventy of 80 (88%) samples stained the white matter tract of human cerebellum and white matter of a frontal lobe biopsy (Fig. 2a and Table 1). Their median hMOG titer was 1:1280 (range 160–20,480). However, 10/80 (12%) samples did not show a positive staining despite their relatively high hMOG titers (median titer 1:640, range 320–20,480). To test whether the observed myelin staining was MOG specific, hMOG antibody-positive samples, used for ex vivo and in vivo experiments (Table 2), were pre-adsorbed against hMOG-transfected cells, resulting in a significant reduction in hMOG titers (data not shown), and in human and rodent myelin staining (Fig. 2b).

**Table 2:** Samples analyzed on murine organotypic brain slices and in the rat EAE model

			CBA			Tissue binding			Slice culture		Rat EAE	
Sample ID	Sex/age (years)	Diagnosis	hMOG titer (1:)	rMOG titer (1:)	mMOG titer (1:)	Human tissue	Mouse tissue	Rat tissue	Total IgG used	MMS (range)	Total IgG used	DMS
MOG 1	F/4	MDEM	2560	160	2560	+	+	+	1 mg/ml	0 (0–1)	n.a.	n.a.
MOG 2	F/7	Rec-ON	2560	160	1280	+	−	+	1 mg/ml	0 (0–1)	n.a.	n.a.
MOG 3	F/8	NMOSD	1280	0	160	+	−	−	1 mg/ml	0 (0–2)	n.a.	n.a.
MOG 4	F/1	MDEM	2560	0	20	+	−	−	1 mg/ml	0 (0–1)	n.a.	n.a.
MOG 5	M/13	Rec-ON	1280	0	20	+	−	−	1 mg/ml	0 (0)	n.a.	n.a.
MOG 6	M/53	NMOSD	5120	0	1280	+	−	−	1 mg/ml	0 (0–1)	10 mg/ml	1
MOG 7	F/67	NMOSD	5120	5120	5120	+	+	+	1 mg/ml	1.5 (0–2)	10 mg/ml	1
MOG 8	F/5	MDEM	640	1280	640	+	+	+	1 mg/ml	0 (0–1)	n.a.	n.a.
MOG 9	F/15	ADEM	320	160	640	+	+	+	1 mg/ml	0 (0–1)	n.a.	n.a.
MOG 10	M/13	ADEM	2560	2560	2560	+	+	+	1 mg/ml	0 (0)	n.a.	n.a.
MOG 11	F/18	NMOSD	2660	0	1280	+	+	−	n.a.	n.a.	10 mg/ml	1
Ctrl 1	M/5	CIS-LETM	0	0	0	−	−	−	1 mg/ml	0 (0–1)	n.a.	n.a.
Ctrl 2	M/10	ADEM	0	0	0	−	−	+	1 mg/ml	0 (0–1)	n.a.	n.a.
Ctrl 3	F/18	MS	0	0	0	−	−	−	1 mg/ml	0 (0)	n.a.	n.a.
Ctrl 4	M/8	ADEM	0	0	0	−	−	−	n.a.	n.a.	10 mg/ml	1
HC 1	F/22	HC	0	0	0	−	−	−	1 mg/ml	0 (0–1)	n.a.	n.a.
HC 2	M/27	HC	0	0	0	−	−	−	1 mg/ml	0 (0)	n.a.	n.a.
HC 3	F/27	HC	0	0	0	−	−	−	1 mg/ml	0 (0–1)	n.a.	n.a.
h8-18-C5			> 40,960	> 40,960	> 40,960	+	+	+	10 μg/ml	3 (2–3)	n.a.	n.a.
m8-18-C5			> 40,960	> 40,960	> 40,960	+	+	+	n.a.	n.a.	5 mg/ml	4
Subcuvia			0	0	0	−	−	−	n.a.	n.a.	10 mg/ml	1
PBS			0	0	0	−	−	−	−	0 (0–1)	n.a.	n.a.

All samples were tested for MOG antibody reactivity on a live cell-based assay (CBA) and on human rat and mouse brain tissue. Slice cultures were evaluated with a median myelin score (MMS) ranging from 0 to 3 (0 = healthy myelin with well-preserved myelin sheath around myelinated axons, 1 = slight MBP degradation and myelin disruption, 2 = partial myelin loss, intermediate to severe MBP damage, 3 = almost complete loss of myelin along myelinated axons and severe MBP loss). Demyelination in the rat EAE model was evaluated with a median demyelination score (DMS) ranging from 0 to 4 (0 = no lesions/slice, 1 = one minor lesion/slice, 0.05–0.2 mm diameter, 2 = two minor lesions/slice or one medium lesion, > 0.2 mm diameter, 3 = two or more medium lesions/slice, 4 = significant loss of myelin)

*Abbreviations*: *MOG 1-11* MOG antibody-positive patients, *Ctrl 1-4* MOG antibody-negative patients, *HC 1-3* healthy controls, *Subcuvia* normal human serum, *h8-18-C5* humanized 8-18-C5 monoclonal MOG antibody, *m8-18-C5* mouse 8-18-C5 monoclonal MOG antibody, *F* female, *M* male, *ADEM* acute demyelinating encephalomyelitis, *CIS-LETM* clinically isolated syndrome longitudinally extensive transverse myelitis, *MDEM* multiphasic demyelinating encephalomyelitis, *NMOSD* neuromyelitis optica spectrum disorders, *rec-ON* recurrent optic neuritis, *MS* multiple sclerosis, *n.a.* not analyzed

In summary, 7 samples showed no binding to human, mouse, or rat myelin tissue, 39 samples were reactive with human myelin tissue only, 1 with rat tissue only, 6 with human and rat tissue, 2 with mouse tissue only, 8 with human and mouse tissue, and 17 with human, mouse, and rat myelin tissue (Additional file 4). Differential tissue binding was associated with increased antibody titers to hMOG, mMOG, and rMOG, but not with demographic or clinical parameters (Additional file 4).

As a control for non-specific antibody binding 20 hMOG antibody-negative samples and healthy controls were included. None of them showed a positive myelin staining in human tissue. Interestingly, 5/20 (25%) hMOG antibody-negative controls showed a positive myelin staining on mouse (1/20, 5%) and rat brain tissue (4/20, 20%) that was not eliminated by pre-adsorption against MOG-transfected cells (Table 1).

### One MOG antibody-positive sample causes complement-mediated myelin damage in murine organotypic brain slices

Ten hMOG-positive samples with sufficient volume for purification and further analysis were used for analysis of pathogenicity on organotypic mouse brain slice cultures. Eight of the 10 samples were reactive to mMOG, and 2 samples had mMOG antibody titers below the cutoff of 1:160 (1:20, Table 2). Six of the 10 samples were reactive to rMOG. Five samples were reactive with mouse, and 6, with rat myelin tissue (Table 2). All samples were positive for hMOG IgG1 antibodies, whereas other IgG subclasses were only present at lower titers (Additional file 5). Sera from three MOG-negative patients with demyelinating CNS disease and three healthy control subjects were also included as additional negative controls (Table 2).

**Fig. 3:**
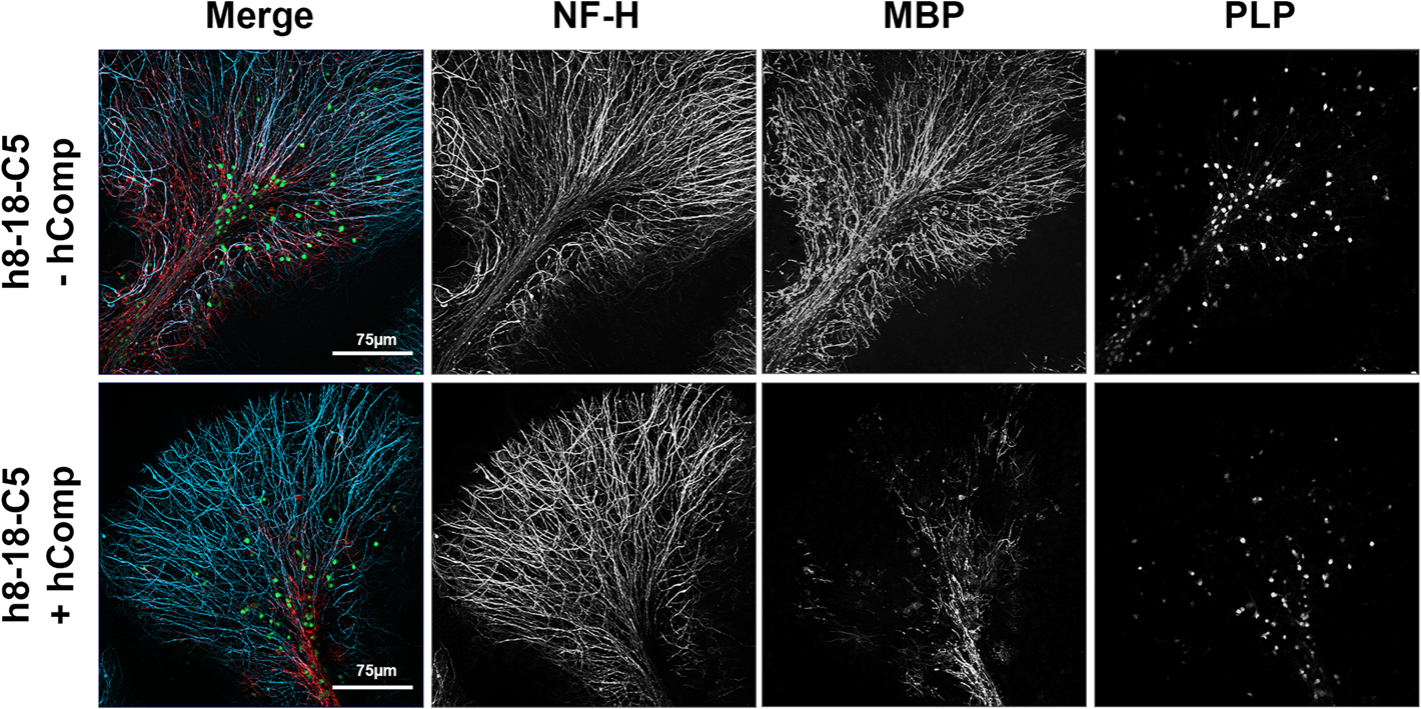
Murine organotypic brain slices incubated with human monoclonal 8-18-C5 with (+ hComp) and without (− hComp) human complement. Cerebellar folia of proteolipid protein (PLP)-EGFP transgenic mice are stained for neurofilament-heavy (NF-H, blue) and myelin basic protein (MBP; red). Confocal images were taken with ×25 objectives

**Fig. 4:**
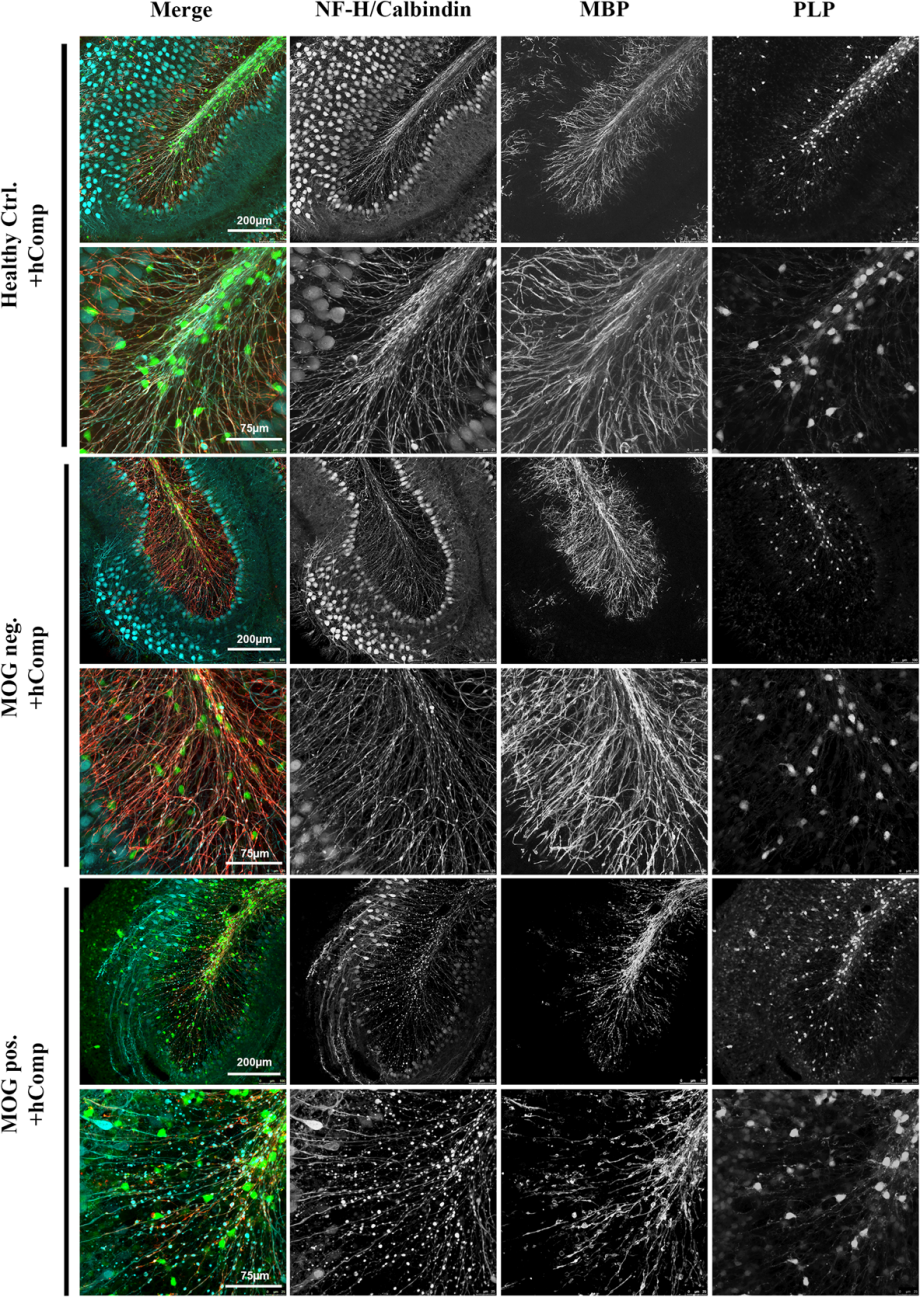
One of 10 human MOG antibody samples causes mild demyelination in murine cerebellar slice culture. Murine organotypic brain slices incubated with total IgG purified from MOG-positive (MOG pos.) and MOG-negative (MOG neg.) human sera with human complement (+hComp) were stained for neurofilament-heavy (NF-H; blue), calbindin (blue), and myelin basic protein (MBP; red). Mild loss of MBP was observed only in the presence of MOG pos. serum (MOG 7) and hComp. Confocal images were taken with ×25 objectives and ×3 digital zoom for higher magnifications

The addition of humanized anti-MOG monoclonal antibody (mAb) 8-18-C5 to the organotypic slices led to fulminant myelin disruption and MBP loss with a MMS of 3 (Fig. 3 and Table 2). Oligodendrocyte death, indicated by loss of PLP-EGFP expression, was not observed following treatment with mAb 8-18-C5. Axonal integrity (NF-H staining) was only partially affected by mAb 8-18-C5 exposure, indicated by regional swellings (Fig. 3). The demyelinating effects were completely complement-dependent (Fig. 3). In contrast, total IgG from only one MOG-IgG-seropositive patient (MOG 7) showed complement-dependent mild pathological effects with a MMS of 1.5 (Table 2, Fig. 4, Additional file 6). Myelin was partly disrupted along axonal swellings, indicated by damage and partial loss of MBP (Fig. 4, Additional file 6). Total IgG from this sample demonstrated the highest titers against hMOG (1:5120), mMOG (1:5120), and rMOG (1:5120) and bound to human, mouse, and rat myelin in tissue sections. All other samples with positive mouse tissue binding patterns (4/9) or reactivity to mMOG (7/9), as well as healthy or MOG seronegative inflammatory controls were negative for antibody-induced pathology (Table 2).

### MOG antibodies show no antibody-mediated pathology in an MBP-T cell-mediated EAE rat model

**Fig. 5:**
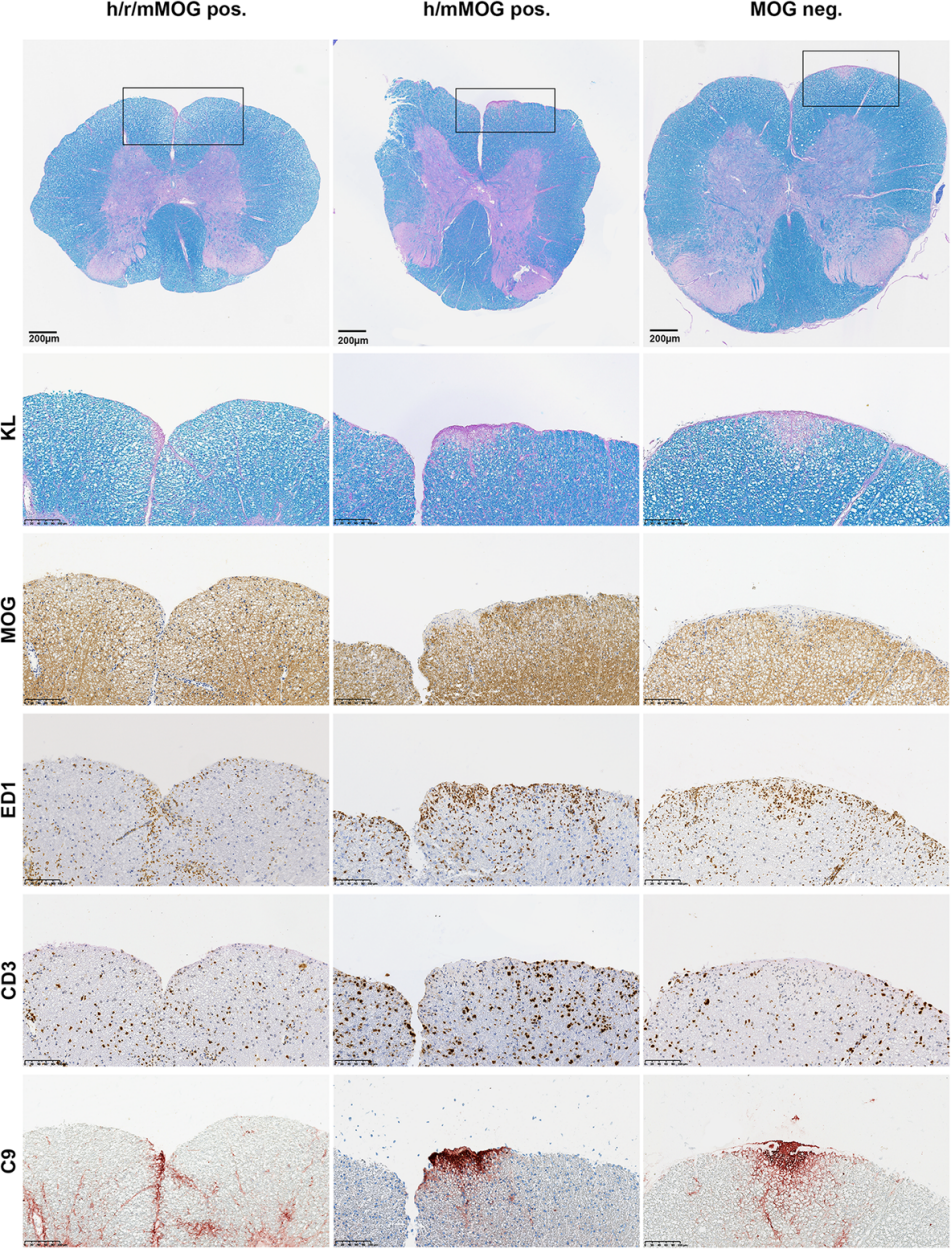
Spinal cord sections of MBP-T cell EAE animals co-injected with patient antibodies. Patient IgG was reactive to either human, rat, and mouse MOG (h/r/mMOG pos., MOG 7); human and mouse MOG (h/mMOG pos., MOG 6); or negative to human MOG (hMOG neg.) tested by CBA KL, Kluver-Barrera (myelin); MOG, myelin oligodendrocyte glycoprotein; ED1, macrophages; CD3, T cells; C9, complement. Spinal cord slices were digitally magnified at 3.8× and highlighted sections at ×20

**Fig. 6:**
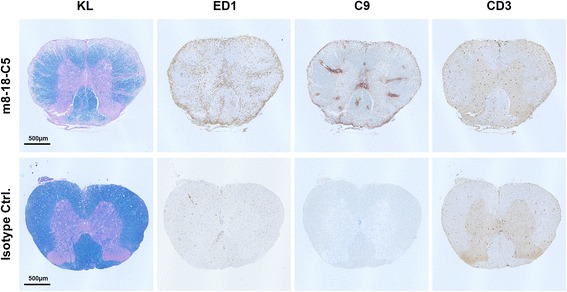
EAE animals co-injected with mouse monoclonal antibody 8-18-C5 (m8-18-C5) vs. isotype control (isotype Ctrl.). Slices were stained with KL, Kluver-Barrera (myelin); ED1, macrophages; C9, complement; CD3, T cells. Spinal cord slices were digitally magnified at ×3.8

We next evaluated two hMOG antibody-positive samples (MOG 6 and MOG 7) with sufficient amount of IgG for co-transfers for pathology in an EAE rat model. MOG 7 was reactive to all tested species and showed a demyelinating effect in murine organotypic brain slices, whereas, MOG 6 was reactive to hMOG and mMOG and showed no pathology on organotypic mouse brain slices. MOG 6 and another MOG IgG-negative sample served as negative controls in the rat EAE model. An additional total IgG sample obtained from a plasma exchange sample was added to this study, only reactive to hMOG (titer 1:2560) and mMOG (1:1280), but not tested on organotypic brain slices (MOG 11, Table 2). Mouse anti-MOG monoclonal antibody 8-18-C5 was used as a positive control. No demyelinating lesions above background levels (DMS = 1, Table 2 and Fig. 5) were seen after injection of MBP-specific T cells with or without MOG 7- or rMOG-negative controls. Further, no effects were noted at a higher IgG concentration (40 mg/ml) and/or after a longer interval (48 h) (data not shown). In contrast, co-transfer of the monoclonal mouse 8-18-C5 at a concentration of 5 mg/ml caused lesions with extensive demyelination, macrophage recruitment, and perivascular complement deposition (DMS = 4, Table 2 and Fig. 6). Following injection, the presence of antibodies to hMOG and rMOG in rat sera were verified by CBA. Titers to hMOG were significantly decreased in rat serum samples compared to original titers (10 mg/ml animals: median titer 1:160, range 1:80–320; 40 mg/ml animals: median titer 1:640, range 1:640). Titers to rMOG were equally low as hMOG titers.

## Discussion

Only a limited fraction of human MOG antibodies reveal reactivity to either mMOG or rMOG by CBA. Reactivity to mMOG (60%) was more frequently seen than rMOG (18%). The present investigation is the largest study on species-specific reactivity of human MOG antibodies conducted so far, confirming results of prior studies with a smaller sample size [40, 41]. Similarly, only a subset of hMOG autoantibody-positive samples was reactive on mouse and rat brain tissue. Only 52% of the mMOG-positive samples but 100% of the rMOG-positive samples (CBA, cutoff ≥ 1:160) showed a positive binding pattern to mouse and rat myelin tissue, respectively. In contrast, 88% hMOG-positive samples bound to human brain sections. Differential binding to mouse, rat and/or human tissue was strongly influenced by antibody titers to mMOG, rMOG, and hMOG. Specificity of the tissue binding was confirmed by pre-adsorbing serum samples with MOG-transfected cells. In case of a positive tissue binding with hMOG-, mMOG-, or rMOG-negative samples, the staining signal could not be diminished by pre-adsorption. In these cases, we cannot differentiate between low-level MOG binding, binding to alternative myelin antigens, or background signal. Staining could be produced by MOG antibodies with epitope specificity to epitopes revealed only after tissue fixation. Conversely, the loss of myelin staining observed for a number of hMOG-positive samples is likely due to the loss of conformational epitopes due to tissue fixation and dehydration. The presence of autoantibodies to alternative myelin and neuronal targets have been reported in the CSF of MS patients [49, 53], and these antibodies may contribute to tissue reactivity both in hMOG-negative and hMOG-positive samples.

The differences in antibody binding to hMOG, mMOG, and rMOG are surprising considering the rather small differences in the amino acid sequences of the three proteins (Additional file 7). A previous study has already established the importance of the C-C′ loop in the recognition of human-specific MOG antibody responses [40] which might also be responsible for the differences in antibody recognition seen in our study. In contrast, antibodies also reactive with mMOG and probably also rMOG mainly recognize the F-G loop, which is also the epitope recognized by the monoclonal antibody 8-18-C5. Further, it is still unknown whether there are differences in the affinity of human antibodies to different MOG epitopes. Whereas it is well known that the affinity of the monoclonal antibody 8-18-C5 is higher than that of other murine MOG antibodies [31], the affinity of human MOG antibodies has not yet been analyzed because human monoclonal MOG antibodies needed for methodological reasons are not available yet.

Serum IgG from only one adult NMOSD patient with high titers to mMOG, rMOG, and hMOG caused obvious myelin loss as evidenced by MBP degradation and signs of axonal disturbances and swellings. Interestingly, this patient had the highest titer anti-MOG IgG of subclass 3 (Additional file 5). Demyelinating effects were only shown by the addition of human complement and could not be replicated without complement. This rare pathology contrasts with other patient samples as well as previous studies that showed minimal complement-mediated pathology using human MOG autoantibodies [38, 39]. Weak complement activation has also been observed in a relapsing encephalomyelitis patient positive for MOG antibodies with complement activation only present at areas of active demyelination [17]. These studies showed that MOG IgG are only to some extent able to activate the complement pathway and therefore mediating pathologic effects; however, our study revealed a major contribution of complement-mediated pathology in this sample, which is in line with several reports observing complement deposition in brain tissue of patients with MOG antibodies [18–20]. Furthermore, this single MOG-IgG-positive patient may target a specific epitope that allows efficient complement activation. Although we cannot completely exclude whether additional autoantibodies in this sample are responsible for the observed pathology, pre-adsorption with hMOG lead to significant reduction of myelin staining, indicating minimal contribution of other autoantibodies against myelin.

While antibodies to MOG are mainly from the IgG1 subtype and therefore able to activate complement in vitro [31, 54] and in vivo [31], the observed effects of hMOG autoantibodies are typically minimal and reversible [38, 39]. Two further antibody injection experiments in rodents did not lead to pathogenic effects in connection with human IgG positive to MOG (A. Saiz, personal communication). In contrast, the monoclonal MOG antibody 8-18-C5 causes severe demyelinated areas in the organotypic brain model [55]. Hence, it appears that MOG antibody-mediated effects in ex vivo mouse models are strongly dependent on high titers to rodent MOG and the high affinity recognition of specific MOG epitopes that facilitate complement activation. Importantly, a recent study has demonstrated that two samples reactive with rodent MOG were able to induce ADCC and CDC in MOG and MBP EAE after injection in the cerebrospinal fluid (Edgar Meinl, personal communication).

In a next step, we were interested whether the anti-MOG antibodies demyelinating in vitro could also cause demyelination in vivo, by antibody-transfer in MBP-specific T cell-induced EAE. This model has already proven to be a suitable animal model to analyze human autoantibody-mediated effects in vivo [28, 56]. In this context, adoptive transfer of MBP-specific T cells trigger cellular and humoral CNS autoimmunity by activating the complement machinery and Fc gamma III receptor-positive cells, thereby boosting ADCC- and CDC-mediated lesions in an *γ*-IFN dependent way in Lewis rats [52]. However, none of the hMOG-positive samples showed any pathological effects in the EAE model, including sample MOG 7 which was reactive to rMOG and caused demyelination in murine organotypic brain culture. One potential explanation for this discrepancy could be low antibody binding affinity in the in vivo rat EAE model. Since antibody titers in rat serum to hMOG and rMOG were relatively low compared to the titers seen in the patients, the concentration of MOG antibodies may have been insufficient to induce pathologic effects. To account for the possibility of an insufficient concentration of antibodies, we also employed a fourfold higher amount of h/rMOG-positive IgG (40 mg); however, we did not observe an increase in demyelination. Similar to the results observed in cerebellar slice cultures, the monoclonal MOG antibody 8-18-C5 applied in milligram amounts led to substantial myelin loss, macrophage activation, and complement deposition.

In addition to CDC and ADCC, human MOG antibodies have recently been shown to trigger disease severity in an EAE model due to the activation of MOG-specific T cells by fostering autoantigen recognition via antigen-presenting cells. MOG antibody-mediated demyelination was not observed in these studies [36, 37]. Thus, hMOG antibody-mediated CDC may not play a significant role in this rodent model. To conclude, the important question whether human MOG antibodies are pathogenic in vivo is still unsolved. Since the complement-mediated demyelination is difficult to study in mice [57], a human MOG transgenic rat model might be needed to analyze effector mechanisms of human MOG antibodies. This would be also very important because cross-reactivity to rodent MOG is not necessarily related with pathogenicity per se. We still cannot exclude, whether MOG antibodies solely reactive to human MOG could also induce demyelination in an animal model more resembling the human species. However, this study showed that cross-reactivity to rodent MOG is a very important factor to analyze potential MOG antibody-mediated effector mechanisms in rodent models.

Our study has several limitations. First, the majority of patients were children, and most of them had a benign monophasic disease course. Only very few adult patients with severe disease courses were included. Indeed, the only sample able to induce pathogenic effects in the organotypic brain slice model was from an adult patient. Second, the purification and injection of total patient IgG may have resulted in insufficient MOG-IgG concentration to induce pathological effects ex vivo and in vivo. One possible approach to circumvent problems with minor antibody concentrations is to directly inject those antibodies intrathecally or intracerebrally. High efficacy has been shown recently, by injecting low doses of 8-18-C5 and complement into the corpus callosum of mice leading to complement-dependent demyelination [58]. Third, as already mentioned above, cross-reactivity to rodent MOG is not necessarily related with pathogenicity in rodents per se and the pathogenic epitope might only be present in human MOG. Forth, we cannot totally exclude, whether additional autoantibodies may contribute to pathology in MOG-seropositive diseases. Future studies may require purified MOG autoantibodies or recombinant antibodies derived from MOG-reactive human plasma cells. Finally, only a subset of samples could be analyzed for the in vivo pathology due to limited amounts of patient material.

## Conclusion

Only a small proportion of antibodies to hMOG are reactive to rodent MOG as analyzed by CBA and tissue IHC. While MOG mAb 8-18-C5 caused vigorous complement-mediated demyelination ex vivo and in vivo, serum IgG from only a single MOG-seropositive patient with high-titer antibodies to mMOG and rMOG was able to induce complement-dependent tissue injury in an ex vivo organotypic brain slice model. The results suggest that high-titer species-specific MOG autoantibodies recognizing unique epitopes on native MOG may be mandatory for CDC in vivo. Future studies should therefore use hMOG antibody-positive samples with high titers and affinity to rodent MOG epitopes or monoclonal MOG antibodies with defined epitope recognition and affinity before testing them in vivo for pathogenic potential.

## Additional files

Additional file 1: HEK293A cells expressing either mouse MOG (mMOG) or rat MOG (rMOG) C terminally tagged with EGFP. (DOCX 2792 kb)

Additional file 2: Demographic and clinical data and antibody reactivity of 80 hMOG antibody-positive patients included in this study according to antibody binding to mouse MOG. (DOCX 87 kb)

Additional file 3: Demographic and clinical data and antibody reactivity of 80 hMOG antibody-positive patients included in this study according to antibody binding to rat MOG. (DOCX 91 kb)

Additional file 4: Demographic and clinical data and antibody reactivity of 80 hMOG antibody-positive patients included in this study according to antibody binding to mouse, rat, and human myelin tissue. (DOCX 101 kb)

Additional file 5: IgG subclass titers of hMOG-positive IgG samples used for organotypic brain slices and EAE experiments. (DOCX 63 kb)

Additional file 6: MBP staining of murine organotypic brain slices shows myelin health status of all MOG-positive samples (MOG 1-10) and MOG-negative control (Ctrl 1) as well as healthy control sample (HC 1) in combination with human complement. (DOCX 6029 kb)

Additional file 7: Comparison of the amino acid sequences of human (hMOG, alpha 1 isoform, Genbank NP_996532.2), mouse (mMOG, Genbank NP_034944.2), and rat (rMOG, Genbank NP_073159.2) myelin oligodendrocyte glycoprotein. (DOCX 134 kb)

## Abbreviations

ADCC: Antibody dependent cellular cytotoxicity
ADEM: Acute demyelinating encephalomyelitis
AQP4: Aquaporin-4
CBA: Cell-based assay
CDC: Complement-dependent cytotoxicity
CIS: Clinically isolated syndrome
CNS: Central nervous system
DMS: Demyelination score
EAE: Experimental autoimmune encephalitis
hMOG: Human MOG
IHC: Immunohistochemistry
mAb: Monoclonal antibody
MDEM: Multiphasic demyelinating encephalomyelitis
mMOG: Mouse MOG
MMS: Median myelin score
MOG: Myelin oligodendrocyte glycoprotein
MS: Multiple sclerosis
NMDAR: *N*-methyl d aspartate receptor
NMOSD: Neuromyelitis optica spectrum disorders
ON: Optic neuritis
rMOG: Rat MOG
TM: Transverse myelitis
