# Supplementary material for: Human antibodies against the myelin oligodendrocyte glycoprotein can cause complement-dependent demyelination
Source: J Neuroinflammation. 2017 Oct 25;14:208. doi: 10.1186/s12974-017-0984-5 (PMC5657084; doi:10.1186/s12974-017-0984-5)

Additional file 1. HEK293A cells expressing either mouse MOG (mMOG) or rat MOG (rMOG) C-terminally tagged with EGFP. DAPI positive cells were excluded for MOG titer evaluation. Red cells are positively stained with hMOG antibody positive IgG reactive to mMOG and rMOG. Cells were analyzed with 20x objectives.


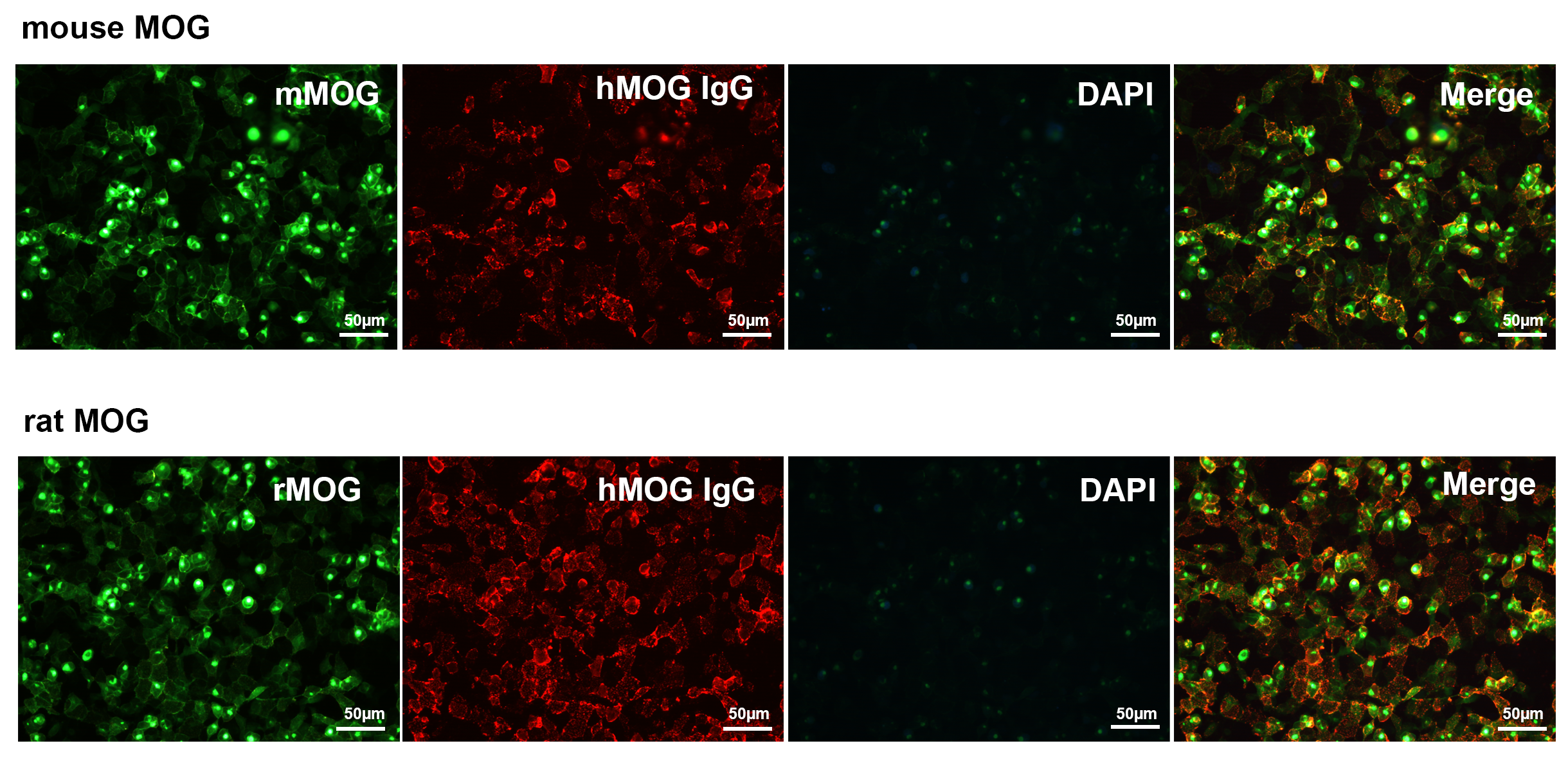

Supplement: Supplementary file 1 — HEK293A cells expressing either mouse MOG (mMOG) or rat MOG (rMOG) C terminally tagged with EGFP. (DOCX 2792 kb) [file 12974_2017_984_MOESM1_ESM.docx]
