# Supplementary material for: Human antibodies against the myelin oligodendrocyte glycoprotein can cause complement-dependent demyelination
Source: J Neuroinflammation. 2017 Oct 25;14:208. doi: 10.1186/s12974-017-0984-5 (PMC5657084; doi:10.1186/s12974-017-0984-5)
Supplement: Supplementary file 2 — Demographic and clinical data and antibody reactivity of 80 hMOG antibody-positive patients included in this study according to antibody binding to mouse MOG. (DOCX 87 kb) [file 12974_2017_984_MOESM2_ESM.docx]

Additional file 2. Demographic and clinical data and antibody reactivity of 80 hMOG antibody positive patients included in this study according to antibody binding to mouse MOG.

|  | **Negative for antibodies to mouse MOG (cut-off ≥ 1:160)** | **Positive for antibodies to mouse MOG (cut-off ≥ 1:160)** | **p-value** |
| --- | --- | --- | --- |
| Number of patients/samples | 32 | 48 |  |
| mMOG antibody titer [1:] ^1^ |  | 640 (160-20480) |  |
| Females | 15 (47%) | 22 (46%) | 0.999 ^2^ |
| Age (years) ^1^ | 6.8 (0.2-71.1) | 7.3 (0.7-67.0) | 0.662 ^3^ |
| Paediatric patients | 29 (91%) | 40 (83%) | 0.511 ^2^ |
| Disease duration (years) ^1^ | 0.1 (0-8.0) | 0.2 (0-15.4) | 0.071 ^3^ |
| Clinical diagnosis at sampling:  ADEM  CIS-ON  CIS-LETM  CIS-multifocal  MDEM  NMOSD  Recurrent ON | 15 (47%)  7 (22%)  3 (9%)  1 (3%)  0 (0%)  4 (13%)  2 (6%) | 22 (46%)  6 (13%)  2 (4%)  1 (2%)  4 (8%)  8 (17%)  5 (10%) | 0.515 ^4^ |
| Recurrent course at sampling | 4 (13%) | 13 (27%) | 0.165 ^2^ |
| hMOG antibody titer [1:] ^1^ | 1280 (160-20480) | 1280 (160-20480) | 0.131 ^3^ |
| Reactive with rMOG | 0 (0%) | 14 (29%) | <0.001 ^2^ |
| rMOG antibody titer [1:] ^1^ |  | 1280 (160-5120) |  |
| Reactivity with brain tissue:  Antibody binding to human myelin  Antibody binding to mouse myelin  Antibody binding to rat myelin  Antibody binding to human + mouse + rat myelin | 28 (87%)  2 (6%)  1 (3%)  0 (0%) | 42 (87%)  25 (52%)  23 (48%)  17 (35%) | 0.999 ^2^  0.001 ^2^  <0.001 ^2^  <0.001 ^2^ |

^1^ median (range), significance of group differences was calculated using ^2^ Fisher’s exact test, ^3^ Mann-Whitney U test and ^4^ Chi square test.

Abbreviations: hMOG = human myelin oligodendrocyte glycoprotein, mMOG = mouse MOG, rMOG = rat MOG, ADEM = acute demyelinating encephalomyelitis, CIS-ON = clinically isolated syndrome, ON = optic neuritis, LETM = longitudinally extensive transverse myelitis, MDEM = multiphasic demyelinating encephalomyelitis, NMOSD = neuromyelitis optica spectrum disorders.
