# Supplementary material for: Human antibodies against the myelin oligodendrocyte glycoprotein can cause complement-dependent demyelination
Source: J Neuroinflammation. 2017 Oct 25;14:208. doi: 10.1186/s12974-017-0984-5 (PMC5657084; doi:10.1186/s12974-017-0984-5)
Supplement: Supplementary file 3 — Demographic and clinical data and antibody reactivity of 80 hMOG antibody-positive patients included in this study according to antibody binding to rat MOG. (DOCX 91 kb) [file 12974_2017_984_MOESM3_ESM.docx]

Additional file 3. Demographic and clinical data and antibody reactivity of 80 hMOG antibody positive patients included in this study according to antibody binding to rat MOG.

|  | **Negative for antibodies to rat MOG**  **(cut-off ≥ 1:160)** | **Positive for antibodies to rat MOG**  **(cut-off ≥ 1:160)** | **p-value** |
| --- | --- | --- | --- |
| Number of patients/samples | 66 | 14 |  |
| rMOG antibody titer [1:] ^1^ |  | 1280 (160-5120) |  |
| Females | 30 (46%) | 7 (50%) | 0.776 ^2^ |
| Age (years) ^1^ | 7.0 (0.2-71.1) | 6.2 (2.6-67.0) | 0.899 ^3^ |
| Paediatric patients | 58 (88%) | 11 (79%) | 0.397 ^2^ |
| Disease duration (years) ^1^ | 0.1 (0-10.2) | 0.1 (0-15.4) | 0.062 ^3^ |
| Clinical diagnosis at sampling:  ADEM  CIS-ON  CIS-LETM  CIS-multifocal  MDEM  NMOSD  Recurrent ON | 30 (46%)  12 (18%)  5 (8%)  2 (3%)  3 (5%)  9 (14%)  5 (8%) | 7 (50%)  1 (7%)  0 (0%)  0 (0%)  1 (7%)  3 (21%)  2 (14%) | 0.728 ^4^ |
| Recurrent course at sampling | 12 (18%) | 5 (36%) | 0.162 ^2^ |
| hMOG antibody titer [1:] ^1^ | 1280 (160-20480) | 2560 (320-20480) | 0.053 ^3^ |
| Reactive with mMOG | 34 (52%) | 14 (100%) | <0.001 ^2^ |
| mMOG antibody titer [1:] ^1^ | 640 (160-2560) | 2560 (160-20480) | <0.001 ^3^ |
| Reactivity with brain tissue:  Antibody binging to human myelin  Antibody binding to mouse myelin  Antibody binding to rat myelin  Antibody binding to human + mouse + rat myelin | 57 (86%)  16 (24%)  10 (15%)  6 (9%) | 13 (93%)  11 (79%)  14 (100%)  11 (79%) | 0.683 ^2^  <0.001 ^2^  <0.001 ^2^  <0.001 ^2^ |

^1^ median (range), significance of group differences was calculated using ^2^ Fisher’s exact test, ^3^ Mann-Whitney U test and ^4^ Chi square test.

Abbreviations: hMOG = human myelin oligodendrocyte glycoprotein, mMOG = mouse MOG, rMOG = rat MOG, ADEM = acute demyelinating encephalomyelitis, CIS-ON = clinically isolated syndrome, ON = optic neuritis, LETM = longitudinally extensive transverse myelitis, MDEM = multiphasic demyelinating encephalomyelitis, NMOSD = neuromyelitis optica spectrum disorders.
