# Supplementary material for: Human antibodies against the myelin oligodendrocyte glycoprotein can cause complement-dependent demyelination
Source: J Neuroinflammation. 2017 Oct 25;14:208. doi: 10.1186/s12974-017-0984-5 (PMC5657084; doi:10.1186/s12974-017-0984-5)
Supplement: Supplementary file 4 — Demographic and clinical data and antibody reactivity of 80 hMOG antibody-positive patients included in this study according to antibody binding to mouse, rat, and human myelin tissue. (DOCX 101 kb) [file 12974_2017_984_MOESM4_ESM.docx]

Additional file 4. Demographic and clinical data and antibody reactivity of 80 hMOG antibody positive patients included in this study according to antibody binding to mouse, rat and human myelin tissue.

|  | **No tissue binding** | **Binding to human tissue only** | **Binding to rat tissue only** | **Binding to human and rat tissue** | **Binding to mouse tissue only** | **Binding to human and mouse tissue** | **Binding to human, rat and mouse tissue** | **p-value** |
| --- | --- | --- | --- | --- | --- | --- | --- | --- |
| Number of patients/samples | 7 (9%) | 39 (49%) | 1 (1%) | 6 (7%) | 2 (2%) | 8 (10%) | 17 (21%) |  |
| Females | 3 (43%) | 15 (39%) | 1 (100%) | 4 (68%) | 2 (100%) | 5 (63%) | 7 (41%) | 0.369 ^2^ |
| Age (years) ^1^ | 5.5 (3.6-18.3) | 6.9 (0.2-71.1) | 7.3 | 7.2 (3.4-22.6) | 5.6, 27.9 | 6.2 (0.7-18.0) | 6.3 (0.9-67.0) | 0.755 ^3^ |
| Paediatric patients | 6 (86%) | 34 (87%) | 1 (100%) | 5 (83%) | 1 (50%) | 7 (87%) | 15 (88%) | 0.867 ^2^ |
| Disease duration (years) ^1^ | 0.1 (0-10.2) | 0.1 (0-8.0) | 0.1 | 2.0 (0-15.4) | 0.2, 1.1 | 0.1 (0-0.1) | 0.1 (0-4.4) | 0.320 ^3^ |
| Clinical diagnosis at sampling:  ADEM  CIS-ON  CIS-LETM  CIS-multifocal  MDEM  NMOSD  Recurrent ON | 3 (43%)  1 (14%)  0 (0%)  0 (0%)  0 (0%)  3 (43%)  0 (0%) | 16 (41%)  11 (28%)  4 (10%)  1 (3%)  1 (3%)  3 (8%)  5 (8%) | 0 (0%)  0 (0%)  0 (0%)  0 (0%)  0 (0%)  1 (100%)  0 (0%) | 2 (33%)  0 (0%)  0 (0%)  0 (0%)  1 (17%)  2 (33%)  1 (17%) | 1 (50%)  0 (0%)  0 (0%)  0 (0%)  0 (0%)  0 (0%)  1 (50%) | 6 (75%)  0 (0%)  0 (0%)  1 (13%)  0 (0%)  1 (13%)  0 (0%) | 9 (53%)  1 (6%)  1 (6%)  0 (0%)  2 (12%)  2 (12%)  2 (12%) | 0.357 ^2^ |
| Recurrent course at sampling | 2 (29%) | 6 (15%) | 0 (0%) | 3 (50%) | 1 (50%) | 1 (12%) | 6 (35%) | 0.343 ^2^ |
| hMOG antibody titer [1:] ^1^ | 640 (320-20480) | 1280 (160-20480) | 1280 | 1280 (320-2560) | 320, 2560 | 5120 (160-10240) | 2560 (320-20480) | 0.054 ^3^ |
| mMOG antibody  mMOG antibody titer [1:] ^1^ | 4 (57%)  320 (160-1280) | 13 (33%)  320 (160-2560) | 1 (100%)  1280 | 5 (83%)  160 (160-1280) | 1 (50%)  320 | 7 (87%)  1280 (160, 2560) | 17 (100%)  1280 (320-20480) | <0.001 ^2^  <0.002 ^3^ |
| rMOG antibody  rMOG antibody titer [1:] ^1^ | 0 (0%) | 0 (0%) | 1 (100%)  320 | 2 (33%)  160, 320 | 0 (0%) | 0 (0%) | 11 (65%)  1280 (160-5120) | <0.001 ^2^  <0.186 ^3^ |

^1^ median (range), significance of group differences was calculated using ^2^ Chi square test or ^3^ Kruskal-Wallis test.

Abbreviations: hMOG = human myelin oligodendrocyte glycoprotein, mMOG = mouse MOG, rMOG = rat MOG, ADEM = acute demyelinating encephalomyelitis, CIS-ON = clinically isolated syndrome, ON = optic neuritis, LETM = longitudinally extensive transverse myelitis, MDEM = multiphasic demyelinating encephalomyelitis, NMOSD = neuromyelitis optica spectrum disorders.
