# Supplementary material for: Human antibodies against the myelin oligodendrocyte glycoprotein can cause complement-dependent demyelination
Source: J Neuroinflammation. 2017 Oct 25;14:208. doi: 10.1186/s12974-017-0984-5 (PMC5657084; doi:10.1186/s12974-017-0984-5)
Supplement: Supplementary file 5 — IgG subclass titers of hMOG-positive IgG samples used for organotypic brain slices and EAE experiments. (DOCX 63 kb) [file 12974_2017_984_MOESM5_ESM.docx]

Additional file 5. IgG subclass titers of hMOG positive IgG samples used for organotypic brain slices and EAE experiments.

| **Sample ID** | **hMOG IgG1** | **hMOG IgG2** | **hMOG IgG3** | **hMOG IgG4** |
| --- | --- | --- | --- | --- |
| MOG 1 | 5120 | 320 | 160 | 80 |
| MOG 2 | 2560 | 0 | 20 | 20 |
| MOG 3 | 2560 | 0 | 20 | 0 |
| MOG 4 | 1280 | 20 | 80 | 0 |
| MOG 5 | 2560 | 40 | 80 | 20 |
| MOG 6 | 10240 | 160 | 80 | 40 |
| MOG7 | 5120 | 160 | 320 | 40 |
| MOG 8 | 320 | 0 | 20 | 0 |
| MOG 9 | 160 | 20 | 40 | 0 |
| MOG 10 | 5120 | 160 | 80 | 80 |
| Median titer (range) | 2560 (1280-10240) | 40 (0-320) | 80 (20-160) | 20 (0-80) |
