# Supplementary material for: Human antibodies against the myelin oligodendrocyte glycoprotein can cause complement-dependent demyelination
Source: J Neuroinflammation. 2017 Oct 25;14:208. doi: 10.1186/s12974-017-0984-5 (PMC5657084; doi:10.1186/s12974-017-0984-5)

Additional file 6. MBP staining of murine organotypic brain slices shows myelin health status of all MOG positive samples (MOG 1-10) and MOG negative control (Ctrl 1) as well as healthy control sample (HC 1) in combination with human complement. Only MOG 7 shows significant myelin loss with blown up myelin along axonal swellings. Confocal images were taken with 25x objectives.


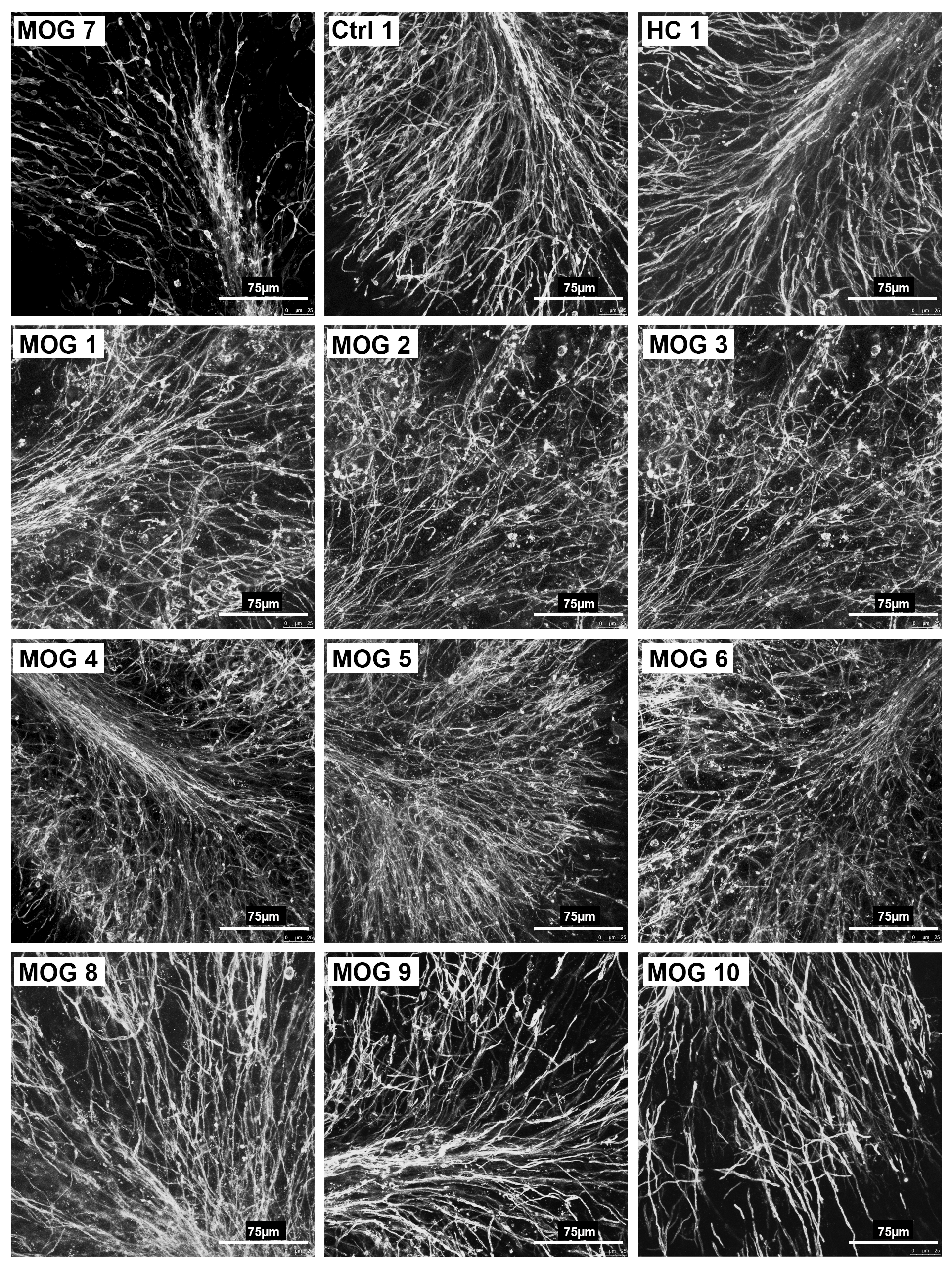

Supplement: Supplementary file 6 — MBP staining of murine organotypic brain slices shows myelin health status of all MOG-positive samples (MOG 1-10) and MOG-negative control (Ctrl 1) as well as healthy control sample (HC 1) in combination with human complement. (DOCX 6029 kb) [file 12974_2017_984_MOESM6_ESM.docx]
