# Supplementary material for: Human antibodies against the myelin oligodendrocyte glycoprotein can cause complement-dependent demyelination
Source: J Neuroinflammation. 2017 Oct 25;14:208. doi: 10.1186/s12974-017-0984-5 (PMC5657084; doi:10.1186/s12974-017-0984-5)
Supplement: Supplementary file 7 — Comparison of the amino acid sequences of human (hMOG, alpha 1 isoform, Genbank NP_996532.2), mouse (mMOG, Genbank NP_034944.2), and rat (rMOG, Genbank NP_073159.2) myelin oligodendrocyte glycoprotein. (DOCX 134 kb) [file 12974_2017_984_MOESM7_ESM.docx]

Additional file 7. Comparison of the amino acid sequences of human (hMOG, alpha 1 isoform, Genbank NP_996532.2), mouse (mMOG, Genbank NP_034944.2) and rat (rMOG, Genbank NP_073159.2) myelin oligodendrocyte glycoprotein.

1 10 20 30 40

hMOG GQFRVIGPRH PIRALVGDE VELPCRISPGK NATGMEVGWY

mMOG GQFRVIGPGY PIRALVGDE AELPCRISPGK NATGMEVGWY

rMOG GQFRVIGPGH PIRALVGDE AELPCRISPGK NATGMEVGWY

-A- -A’- ---B---- ---C--

50 60 70 80

hMOG RPPFSRVVHL YRNGKDQDGD QAPEYRGRTE LLKDAIGEGK

mMOG RSPFSRVVHL YRNGKDQDAE QAPEYRGRTE LLKETISEGK

rMOG RSPFSRVVHL YRNGKDQDAE QAPEYRGRTE LLKESIGEGK

- --C’- -- -C’’ -D- - --

90 100 110 120

hMOG VTLRIRNVRF SDEGGFTCFF RDHSYQEEAA MELKVEDPFY

mMOG VTLRIQNVRF SDEGGYTCFF RDHSYQEEAA MELKVEDPFY

rMOG VALRIQNVRF SDEGGYTCFF RDHSYQEEAA VELKVEDPFY

-E--- ----F-- -- -----G ------ --

130 140 150 160

hMOG WVSPGVLVLL AVLPVLLLQI TVGLIFLCLQ YRLRGKLRAE

mMOG WVNPGVLTLI ALVPTILLQV SVGLVFLFLQ HRLRGKLRAE

rMOG WINPGVLALI ALVPMLLLQV SVGLVFLFLQ HRLRGKLRAE

transmebrane and intracellular region -----

170 180 190 200

hMOG IENLHRTFDP HFLRVPCWKI TLFVIVPVLG PLVALIICYN

mMOG VENLHRTFDP HFLRVPCWKI TLFVIVPVLG PLVALIICYN

rMOG VENLHRTFDP HFLRVPCWKI TLFVIVPVLG PLVALIICYN

---------- ---------- ---------- ----------

210

hMOG WLHRRLAGQF LEELRNPF

mMOG WLHRRLAGQF LEELRNPF

rMOG WLHRRLAGQF LEELRNPF

---------- --------

Amino acid sequences were compared using Clustal Omega (SOAP) software (<http://www.ebi.ac.uk/Tools/webservices/services/msa/clustalo_soap>). Differences between hMOG, mMOG and rMOG are highlighted in red.

Below the alignment, the secondary structure elements of mMOG are shown with ß-sheets in the extracellular immunoglobulin domain labeled A-G and the C-terminal transmembrane and intracellular regions highlighted in blue. The FG-loop and neighboring residues that represent the center of the epitope recognized by monoclonal antibody 8-18C5 are highlighted in yellow. The alignment was adapted from Mayer et al. 2013 [[40](#_ENREF_40)].
